# Corrigendum: *Chlamydia pneumoniae* CdsL regulates CdsN ATPase activity, and disruption with a peptide mimetic prevents bacterial invasion

**DOI:** 10.3389/fmicb.2021.726518

**Published:** 2021-10-12

**Authors:** Chris B. Stone, David C. Bulir, Connor A. Emdin, Ryan M. Pirie, Elisa A. Porfilio, Jerry W. Slootstra, James B. Mahony

**Affiliations:** ^1^Michael G. DeGroote Institute for Infectious Disease Research, Faculty of Health Sciences, Department of Pathology and Molecular Medicine, McMaster University, Father Sean O'Sullivan Research Centre, St. Joseph's Healthcare, Hamilton, ON, Canada; ^2^PepScan Presto, Epitope Mapping, Lelystad, Netherlands

**Keywords:** type III secretion, peptide mimetic, *Chlamydia*, ATPase, Pepscan

In the original article, there was a mistake in Figure 5 as published. Two lanes of a gel were fused together to decrease the size of the figure and improve clarity, when in fact they were not run in side-by-side lanes on the gel. The corrected Figure 5 appears below.

**Figure 5:**
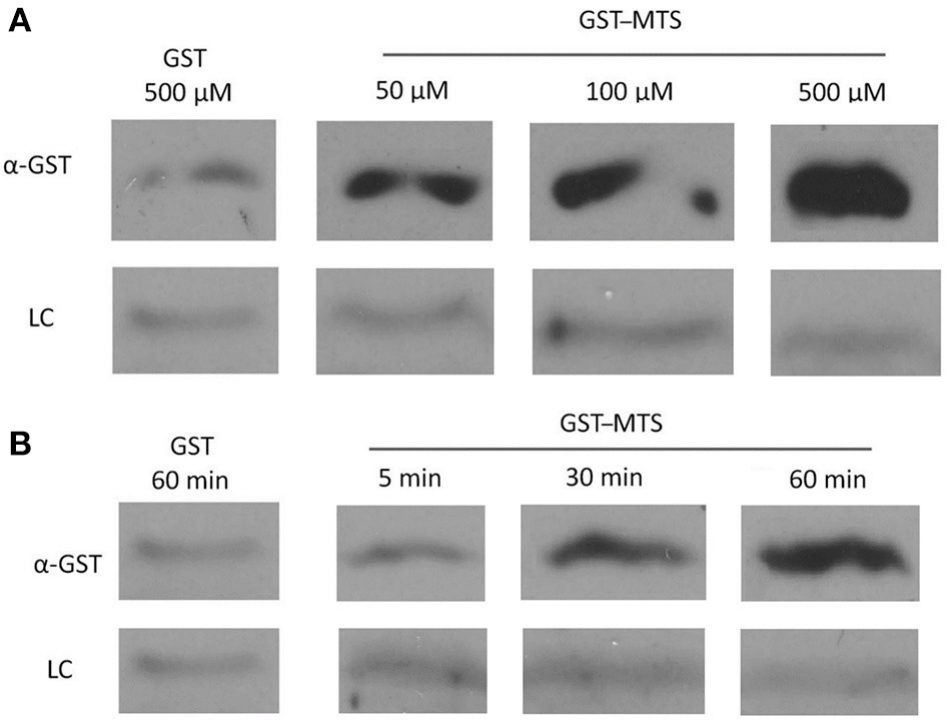
Uptake of a GST-Membrane transport signal (MTS) fusion protein into EBs. EBs were purified on a discontinuous gastrografin gradient and collected by centrifugation to ensure the use of a homogenous EB mixture. The GST alone and GST–MTS protein was expressed in *Escherichia coli* cells and purified using glutathione beads. The EBs were then incubated with either GST or GST–MTS, trypsinized for 30 min to ensure that all extracellular GST or GST–MTS was degraded, and examined by α-GST Western blot for the presence of intracellular GST-tagged protein. CdsL, an intracellular type III secretion protein, was used as a loading control (LC). **(A)** Time-course for incubation of EBs with GST–MTS for 5, 30, or 60 min. GST control at 60 min displayed very little protein while GST–MTS at 5, 30, and 60 min had increasing amounts of GST–MTS present within the EBs. **(B)** Dose–response of EBs incubated with GST–MTS at 50, 100, and 500 μm. GST control at 500 μm displayed very little protein while GST–MTS accumulated within the EBs as the concentration increased. CdsL is shown as a LC.

The authors apologize for this error. Given this correction to Figure 5, internalization of the peptide is now unclear and we do not have strong evidence to suggest that the peptide is affecting an intracellular protein interaction. Further studies are required to support this hypothesis. The original article has been updated.

## Publisher's Note

All claims expressed in this article are solely those of the authors and do not necessarily represent those of their affiliated organizations, or those of the publisher, the editors and the reviewers. Any product that may be evaluated in this article, or claim that may be made by its manufacturer, is not guaranteed or endorsed by the publisher.

